# Awareness of users and motivational factors for using new psychoactive substances in Belgium

**DOI:** 10.1186/s12954-020-00393-0

**Published:** 2020-07-25

**Authors:** Sarah Simonis, Michaël Canfyn, Anton Van Dijck, Tina Van Havere, Eric Deconinck, Peter Blanckaert, Lies Gremeaux

**Affiliations:** 1 D.O. Public Health and Epidemiology, Sciensano, Brussels, Belgium; 2 Present address: Faculté des Sciences Sociales – Service de Criminologie, Quartier Agora, Bâtiment B31, Place des Orateurs 1, 4000 Liège, Belgium; 3 D.O. Chemical and Physical Health Risks, Sciensano, Brussels, Belgium; 4 University College Ghent, Ghent, Belgium

**Keywords:** New psychoactive substances, Qualitative research, Motivations, Profiles, Awareness

## Abstract

**Background:**

Few data on motivations for using new psychoactive substances (NPS) are available. However, the cost, the legal status, and their accessibility through channels like internet contributed to the popularity of NPS. The objective of this article are first to gain a deeper understanding of the culture surrounding NPS in Belgium and second to define the awareness of the users concerning the content of the NPS they are consuming.

**Methods:**

Snowball sampling and partners in the drug demand reduction field were used as a gateway in order to reach a heterogeneous study population. In total, 45 users were recruited and in-depth interviews were conducted. The personal experiences of NPS users and their needs for support along the continuum of care were explored through an interview guideline, while subjects were given the opportunity to deposit a NPS sample for forensic analysis in a recognized laboratory.

**Results:**

A diversity of profiles was found among NPS users but also a wide diversity in the motives to consume NPS: personal reasons such as pleasure, mind exploration, being connected to others, or out of curiosity, but also external reasons such as price, accessibility or the specific effects procured by certain NPS. The results showed as well that a majority of NPS users seem to be aware of the substances they are using.

**Conclusion:**

Understanding the motivations of use is of importance to determine which type of NPS targeted interventions are adapted to different profiles of users.

## Background

The fast-evolving nature of the drug market and especially the new psychoactive substances (NPS) phenomenon is reflected in the large number of substances being produced, distributed, marketed, and detected [1–4]. But also in their diversity and the speed by which the chemical structures of those substances are changed or updated, for example, as a response to changing legislation. Furthermore, the internet has played a significant role by facilitating the acquisition of NPS in an international market [5]. By the end of 2016, the European Monitoring Centre for Drugs and Drug Addiction (EMCDDA) already identified over 620 different NPS present on the European drug market [6]. NPS are not a new phenomenon in term of design [7]; however, the speed by which these substances are produced and emerge on the international market, in particular via internet, has never been seen before, and contributes significantly to the increased NPS availability [8]. Therefore, the “Web 2.0 allowed the spread of an anarchic free-market world in which drug legislation is being outpaced by chemistry and technology” [9]. Two studies confirm that the use of NPS is also influenced by the supply reduction and substance displacement, as well as the quality of the classic substances available on the market [10, 11].

Few data on the motivating factors of the NPS consumers are available. Presumably, the lower cost, temporary legal status, and (online) accessibility helped increase the popularity of NPS [12, 13], in addition to users turning to NPS for a variety of reasons including cognitive enhancement, creativity, pleasure, or self-medication [5, 14]. Furthermore, motives may vary depending on the specific class of substances and the effects desired by NPS users. For example, factors of motivation for using stimulants include enhancement and facilitation of social situations; the use of hallucinogens and dissociative drugs is more connected to self-exploration; the main motives for the use of opioids are coping with everyday life situations, physical, or emotional issues such as pain, anxiety, and sometimes addiction [15]. In summary, the motivations to use NPS differs not only according to the type of NPS but also according to the context. That is the findings of a study realized in 6 countries [16]. Based on a new measurement tool, called the new psychoactive substance use motives measures (NPSMM), five intrinsic motivational factors: enhancement, social, conformity, coping, and expansion have been developed in order to gain a better understanding of the motives to use NPS. Despite a variability across the different countries, tendencies emerged. While stimulant empathogens are connected to enhancement and social motivations, psychedelics, and dissociatives are intertwined with motivational factors such as expansion. The use of dissociatives is also linked to coping motives, with associated factors such as stress, anxiety, and a higher frequency of use. However, these motivational factors tend to be variable depending on the different groups of users [16].

In Belgium, in the period before September 2017, there was no legislation in existence specifically created to tackle the NPS phenomenon. However, on September 26, 2017, a Royal Decree was published in the Moniteur Belge/Belgisch Staatsblad, aimed at controlling as many NPS as possible, by including the core structure for several classes of substances in a generic law. This means that in Belgium all NPS are now illegal. Available data on the prevalence and use of NPS are scarce. In the Belgian health survey (general population), the use of NPS was only questioned in 2013 for the first time. That year, 0.1% of the respondents reported last year use of “legal highs” [17]. After an increasing trend, a decrease in the number of NPS detected by the Belgian Early Warning System Drugs (BEWSD) on Belgian territory was observed since 2016. Forty-nine NPS were detected in 2017 and 104 NPS in 2016, cathinones being the most popular substances detected. Before 2015, hardly any synthetic opioids were reported or detected in Belgium. However, since then, several deaths were caused by the consumption of NPS, with most of these deaths instigated by consumption of NPS opioids. Non-fatal intoxications were reported to the BEWSD related to the consumption of NPS as well [18].

The first main objective of this article is to gain a deeper understanding of the culture surrounding NPS use in Belgium, including the profile(s) of NPS users, the substances they use(d), and the meaning of their use. Therefore, the experiences of NPS users as well as motivations on NPS use were key elements. Due to serious adverse effects related to the use of these products [5], e.g., the combination of different active substances, wrongly assumed dosages and potential presence of toxic impurities, the identification of the NPS that are being used by recruited NPS users is of major interest as well. Therefore, the second objective is to define to what extent NPS users are aware about the composition of the substances they use. To this end, the ideas and presumed knowledge of users on a submitted NPS product was compared to a subsequent analytical screening in a toxicological laboratory. In this study, the simplified version from the EMCDDA was chosen to define a “new psychoactive substance”: a new narcotic or psychotropic drug, in pure form or in preparation, that is not controlled by the 1961 United Nations Single Convention on Narcotic Drugs or the 1971 United Nations Convention on Psychotropic Substances, but which may pose a public health threat comparable to that posed by substances listed in these conventions [19].

## Methods

Eligible participants to the study had to meet two inclusion criteria: a minimum age of 18 years or older at the time of the interview and participants had to have used NPS at least twice in the last 12 months prior to the interview. As GHB or ketamine consumption was not considered to ensure NPS substance diversity, users should at least have used one other NPS other than Ketamine or GHB in the previous year. Both are examples of long-existing drugs, their newly emerged usage patterns as “clubdrugs” are the reason why ketamine and GHB are often included into the body of NPS in research, as well as in this study [6].

Study participants received a financial incentive of 20 EUR. In-depths interviews were organized; they took from 35 up to 90 min and were performed at a location agreed upon by both respondent and researcher, such as the researcher’s office, a bar, the user’s home, and treatment centers.

In order to recruit a diverse and heterogeneous sample of NPS users, a snowball sampling technique was applied relying on partners of the drug demand reduction field as a primary gateway of the zero-chain (e.g., treatment centers, harm reduction, and prevention initiatives, syringe exchange program, peer-support). However, within the foreseen recruitment period, too little respondents were reached, requiring a change in protocol in order to obtain the desired number of study participants. Additional gateways were installed, including health promotion services, colleges, and universities, art schools, men having sex with men (MSM) associations, medical houses, the network of the Belgian Early Warning System Drugs, and internet fora such as psychonaut, psychoactif, Avengers, the Hub, and Reddit. The role of these partners was to help the researchers’ team to get in touch with the different type of NPS users by relaying information on the study, allowing researchers to present the project during gathering with users, distributing flyers during some activities…

The use of these different sources enabled the identification of a new “zero-chain” for the snowball sampling, and helped to establish some degree of confidence when interacting with users [20].

In total forty-five in-depth interviews were conducted in Belgium on a national level: 28 in the Dutch-speaking community and 17 in the French-speaking community. A trial interview was done in June 2017, and the actual interviews started in September 2017. Two researchers performed the interviews, so users could be interviewed in their own language (French or Flemish).

To ensure reproducibility and consistency of the results between the two researchers, interview guidelines were established based on the literature available on the topic. [21–27]. These guidelines were divided into three parts:

i) The socio-demographic assessment
ii) The substances used (both classic and novel substances used during the last year)
iii) The further in-depth interview constructed around 5 major themes:
i) NPS terminology
ii) Motivation and context of use
iii) Mode of use and harm reduction strategies/social control
iv) Attitude towards NPS (price, availability, purity, friends, legal status…) and personal consequences
v) Knowledge and health care needs

Interviews were audio recorded using a tape recorder and fully transcribed verbatim. Subsequently, thematic analyses were conducted using NVivo 10, a software for qualitative analysis. The analyses were performed by the two researchers who performed the interviews, speaking Dutch and French respectively. In order to increase the reliability and validity, a final coding tree was elaborated on and reviewed by both researchers [28]. In order to underline specific themes and meanings that may have been manifest or latent [29], the initial structure of the interview guideline was used to create the different categories for the coding tree. However, during the coding of the interviews, the tree was adapted and enlarged using new nodes and sub-nodes, when a new and significant theme was detected. Each change was communicated and validated by the other researcher.

At the end of each in-depth interview, the recruited NPS users had the opportunity to submit one to three NPS samples they had used before, to be analyzed in the Medicines Laboratory at Sciensano. Users were asked about the presumed identity of the sample, their habits on retrieving information on substances in general as well as the source of acquisition of the aforementioned sample. These samples were photographed, put in a sealed envelope together with an analysis form filled in by the researchers. Finally, the sample was transported legally to the laboratories of Sciensano by the researcher or by a qualified person, a license from the federal agency for medicines and health products was used to this end. Feedback of the results of scientific identification of the samples’ contents was provided to the users by phone or mail, directly after receiving the results from the laboratory. An informed consent was also signed by users in order to correctly inform them on the procedure and the personal data protection. The study was approved by the Ethics Committee of the University of Ghent (Faculty of Psychology and Pedagogy).

At the laboratory, depending on the nature (pill or powder) the sample was crushed to obtain a powder or immediately dissolved in methanol for further analysis. In the case of liquid samples, sample preparation was performed using extraction with dichloromethane. These sample preparation steps were performed in a security cabinet. After sonification of the obtained solutions using an ultrasonic bath and filtration, samples were analyzed using gas chromatography hyphenated with a single quadrupole mass spectrometer (GC/MS, Agilent Technologies 7890A and 5975). When a signal was detected, the mass spectrum was compared using several different spectral libraries including NIST, the Cayman Forensic library, and some in-house libraries. When standard reference substances were readily available, quantification was also performed using GC/MS, ultra-violet analysis, and/or liquid chromatography.

This procedure for identification of illegal substances is accredited by BELAC (ISO17025) and is subject of the OMCL certification (ISO17025) of the laboratory, issued by the European Directorate for Quality of Medicines (EDQM) part of the Council of Europe.

## Results

### Description of participants

Most NPS users (82%) recruited in this study were between 25-45 years, with an average of 33.47 years and are predominantly men (69 %) (Table 1). Few respondents were included with ages over 45+. When assessing the different categories of NPS types as reported by the respondents during the interviews, stimulants seems the most popular class of substances. Dissociatives are frequently mentioned, followed by psychedelics and depressants. The category cited the least were opioids, including one unintentional use.

**Table 1:** Characteristics of respondents in Belgium and reported use of NPS

**Number of respondents by gender**	
Women	14
Men	31
**Number of respondent by age category**	
18-24	5
25-30	11
31-35	14
36-45	12
45+	3
**Category of most used NPS (last year)**	
Stimulants	28
Dissociatives	23
Psychedelics	13
Empathogens	7
Depressants	14
Cannabinoids	6
Opioids	5

### User’s knowledge and conceptions about NPS samples

Table 2 is showing a comparison between the description by the participants of the samples collected from them and the substance identity as determined during the laboratory analysis. Thirty-seven samples were deposit and analyzed in the laboratory. The most popular substances identified in this study were 6-(2-aminopropyl)benzofuran (6-APB) and mephedrone (4-methylmethcathinone, 4-MMC). Another popular substance was 4-fluoroamphetamine (4-FA). In general, the identity of the substance as identified by the analytical laboratory corresponded to the identity as suspected by the user for a small majority of submitted samples (63%). However, two things are worth mentioning. First of all, for several samples, the researchers were not able to establish the identity of the substance using the sample description presented by the users. For example, suspected identities included “yellow speed,” “spice,” liquid XTC, “heroin analog,” “cocaine analog,” and “purple syrup” (Table 2 should be placed here).

**Table 2:** Presumed identity of the samples and laboratory results

N°	Presumed identity given by NPS users	Identification by laboratory analysis^**a**^
1	“Speed jaune”	MDMA
2	Spice blend “Cherry Haze”	AB-Fubinaca
3	Spice blend “Blueberry burst”	JHW-018 + AB-Fubinaca
4	Spice blend “Tropica 2.0”	JHW-018
5	Pill (user did not know the name)	Negative
6	3-MMC	4-MMC
7	6-APB	6-APB
8	“Liquid ecstasy”	GHB + MDMA + Cafeine
9	Methylone	Alpha-ethylaminohexanophenone
10	Methoxetamine	Ketamine
11	4FA	MDMA
12	6-APB	2-APB
13	25i-NBOMe	2C-C-Nbome
14	Heroin analog	Negative
15	Ketamine	Ketamine
16	2C-E	2C-E
17	GBL	GBL
18	Cocaine-analog	Cocaine
19	3MMC	2-methylethcathinone
20	6APB-25NBOMe-3MMC	2-APB
21	3MMC	3MMC
22	Hexen	Hexen (Alpha-ethylaminoexanophenone)
23	4-FA	4-FA (4-Fluoroamphetamine)
24	3 MeO PCP	3 MeO PCP (3-METHOXY PCP )
25	Eth_Lad	Negative
26	Hexen	Paclobutrazol
27	DSK	DSK (Deschloroketamine)
28	DSK	Pyrovalerone
29	Ketamine	Ketamine + MDMA
30	“Purple syrup”	Negative
31	3-MMC	4methylmethcathinone + dimethyl sulfon
32	3.4 DDMC	3.4 DDMC (3.4-Dimethylmethcathinone)
33	4-MMC	4-MMC (4methylmethcathinone)
34	Ketamine	Ketamine
35	4-FA	3-FA (3-Fluoroamphetamine)
36	6-APB	6-APB
37	Al-Lad	Negative

^a^The name between bracket is the official name of the substance identified by the laboratory

In addition, several substances could not be identified by the laboratory using the available analytical techniques, further complicating the matter. The problem of positional isomers cannot be underestimated: several NPS analogs exist where the only difference with neighbor analogs is the position of some substituents, for example, on a phenyl ring. Known examples include the benzofurans 5-APB and 6-APB, or the cathinones 3-MMC and 4-MMC. The technique used to identify the molecules in the laboratory do not enable straightforward determination of these positional isomers; as a result, the predominant isomers were selected for further analysis.

Most respondents are aware of the specific character of each NPS substance, and they could provide a correct definition of the NPS concept: newly designed molecules that mimic the desired effects of classic illicit drugs. Notions related to molecular modifications, falsification, and the connection with the legal aspects are well-known as well. Other notions that were commonly mentioned are “synthetic substances,” “non-traditional substances,” or even “a kind of substitute to classic illicit drugs.” Although almost all users know that some NPS was created in order to circumvent the illegal character of the classic illicit drugs, they seem hardly informed on the current legal status of NPS in Belgium (generic law implemented in September 2017). Many users are not able to distinguish which molecules fit the NPS category and seem to think that NPS only cover certain categories such as stimulants or empathogens, while not being aware that NPS can cover any category including for example opioids and depressants. Moreover, it is not clear for users which specific substances are an NPS (such as methoxetamine or synthetic cannabinoids).

The results of the qualitative interviews are described in the following section with an emphasis on the profiles, contexts, and motivations of use.

### Between experienced and occasional users: various profiles of NPS users

The study population is characterized by a great diversity and heterogeneity of the respondents. Despite this, categories of NPS users could be distinguished. These categories are based on the qualitative interviews and established on the following criteria: knowledge on NPS substances themselves as well as harm reduction strategies, the context of NPS consumption and potential problematic use. The term “problematic use” was defined according to NPS users’ opinions, and was used by study participants to describe their own consumption. In total, three user categories could be determined using this approach: experienced users, underprivileged users, and occasional users.

- *The experienced users*. Knowledge and experiences are the main criteria to describe them. They have a true expertise on the different products, effects, and consume several different NPS substances in order to explore and satisfy their own curiosity. According to the respondents, social context is a favored motivation, but also reasons such as personal exploration or creativity are not excluded. The majority of them use several NPS on different occasions and have had experience with different NPS classes (e.g. cathinones, psychedelics, empathogens and opioids). Their access to information (via internet, friends) and harm reduction strategies seems easier than it is for the other groups. A minority of them mentioned a problematic use at some point in their lives. The NPS use can be occasional or more intense.
- The underprivileged users. Here, the use of NPS is more ancillary to classic illicit substances. Moreover, it is most of the time intertwining with classic drug use. Focusing on their NPS use and using only one type of substance is the rule. They report the use of NPS limited to specific products (e.g., ketamine, GHB, fentanyl). This category of users seems less informed on the products and their access to information is limited. Despite this lack, some of them are however aware of harm reduction strategies. For most respondents of this group, the use of substances is problematic regardless of the type of drug used. This underprivileged group frequently attends low-threshold care (ambulatory) or needle and syringe exchange program (NEP).
- The occasional users. In this group, from the respondents, the NPS consumption ensues mainly in the nightlife settings and/or in a specific social context; the use is particularly controlled, meaning that the use of NPS is clearly occasional, restricted to specific situation (e.g., only the weekend) and limited to 2 or 3 products. The knowledge on NPS seems better than for the underprivileged group but they are not as experienced as the first group. They have adequate access to information and harm reduction strategies.

The different gateways provided access to the different groups of users. For example, syringe exchange program leads us to the group of deprived users, while experienced and occasional users were notably found through internet fora. Harm reduction and prevention initiatives played a role for all types of users, depending on the activities offered by them. Activities linked to nightlife were a channel to enter in contact with occasional and experienced users, low-threshold structures to get in touch with a more underprivileged group, and peers with the experienced one.

### Multiplicity of contexts and motivational factors

**Fig. 1:**
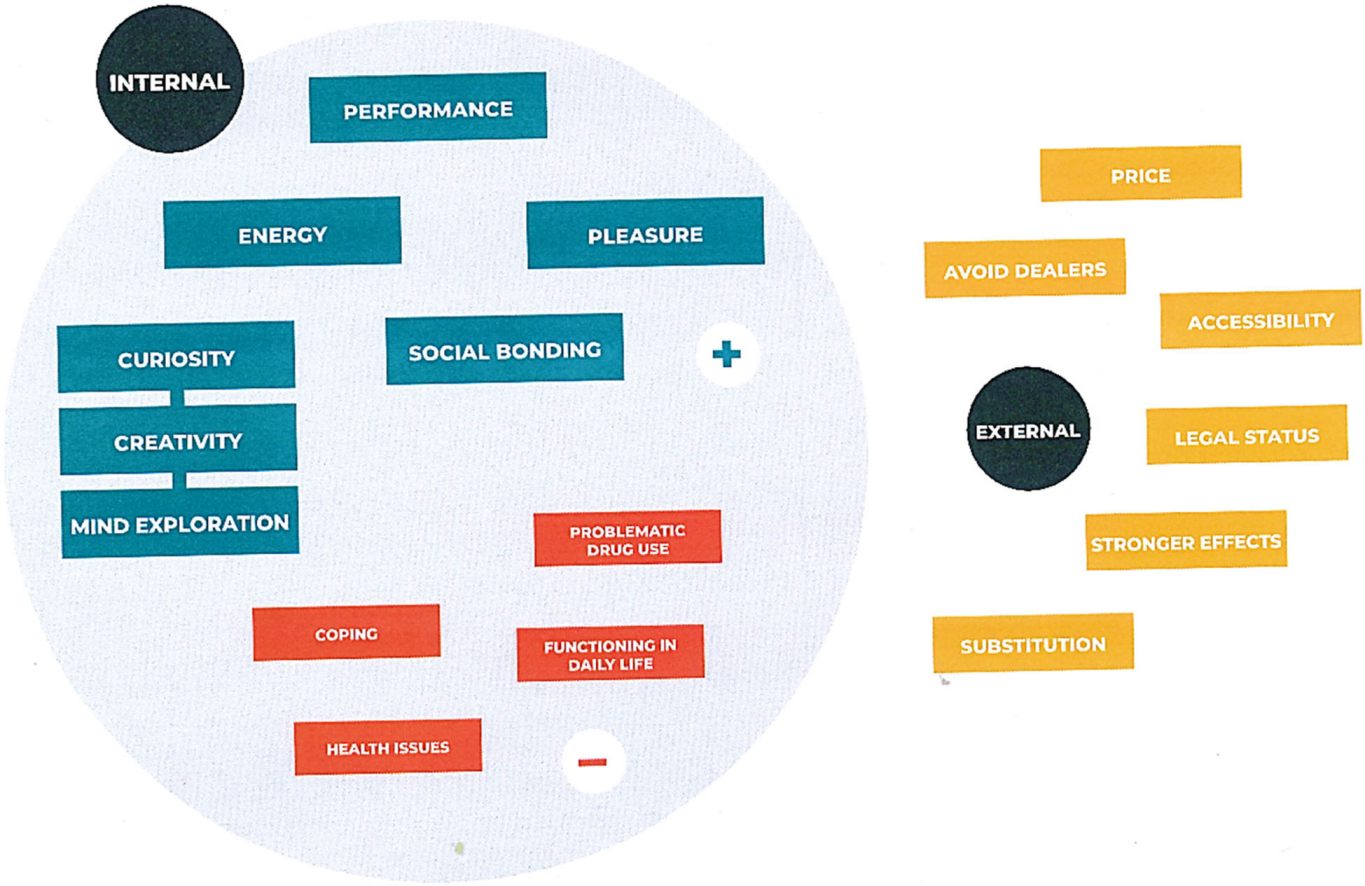
Visualization of motivational factors by type of motivations for using NPS

From the interviews, it can be concluded a first distinction between the internal and the external motivations for using NPS [15, 23, 30]. Also, still based on the qualitative data, a second distinction can be clearly realized within the internal categorization using a division between the positive and the negative internal motivations. We provide an overview and explanation of the eight principal motives in the text below. These motivational factors are listed in order of importance in each category of motivations, and Fig. 1 illustrates this (Fig. 1 should be placed here with colors).

#### Positive internal motivations

##### Pleasure

According to the respondents, this motivation was the most cited for using NPS. This theme is strongly connected to the nightlife context, namely to party, dance, have more energy, connect with people, loosen up, “get high,” and lower inhibitions. The social context is a key element for this theme, as well as the connection with others and the social bonding. Associated products mainly are the stimulants and the empathogens though also dissociatives and psychedelics were also mentioned. For some users, NPS may replace alcohol during the party aiming to stay up all night while remaining energetic or being more extroverted and having a sense of well-being. Moreover, using NPS with friends at home is also a favored setting. In this particular situation, social bonding with friends meant talking, having fun with friends but can also lead to sex. This “Pleasure” category is usually linked to the category “Connection and social bonds” but also to the “Energy” category. This factor of motivation is present for the 3 types of users though a little bit less for the underprivileged users.

Why I do MDPV? The thing is, I’ve always been interested in stimulants and when I convert it and smoke it … Well, you get an instant sort of gigantic arousal, even lechery; a giant flash. And that is pure pleasure, sheer bliss. In first instance, you are completely out of your head and you simply enjoy the ride. (Male, 32)

##### Energy/performance

In this category, different components can be highlighted. While having enough energy in the nightlife context was cited, the acquirement of energy in everyday’s life was also mentioned. For instance, NPS were used to enhance performances in a job environment or even in a study context. In connection to these last two arguments, NPS can be used to cope with the day-to-day context to cope with daily tasks or difficulties. For this reason, and according to the respondents, NPS use results in later problematic use. This motive concerns more the experienced and the occasional users.

Cause without my dope, doing that job? I don’t think so… I wouldn’t even consider getting up. Of course not. Because I know, I could keep this up for a week or so but after that…? No, of course not. It also helps to listen to the small talk of customers; something you have to do, something which makes ‘em come back. When I would be sober, I would probably say: Sure madam, you are totally right but you also told me this last week… And on gear, my reaction is like: Really? Blablabla… Then the lady leaves and is happy and I am happy. I mean, not happy but I do my job really well and it helps me with it. Sure. (Male, 57)

##### Curiosity

This classification includes, on the one hand, the curiosity in a global context, namely, an interest to try something new and discover new sensations. On the other hand, especially for the more experienced users, the curiosity is related to the wish to deepen the exploration of substances or a spiritual exploration of themselves, such as discover new effects, feel the difference between the types of drugs used but also between NPS and classic illicit drugs. For some experienced users, experimenting NPS can be compared to creating and expanding a collection, an assortment of senses, trying something rare or more dangerous as well as pushing back their own limits. Experienced users are more involved in this motivation.

(…) I started to visit internet fora, mainly psychonaut.com, I think I have worked for a couple of years as a forum moderator. When reading my old posts again I realized again that there were really a lot of new products easily available, which made me curious. It was an almost scientific curiosity of knowing which were the existing products, so I became interested a lot in psychopharmacology, to become more informed about the effects before trying the substances. (Male, ± 30)

##### Connection and social bonds

Two dimensions can be considered in this category. First, the sense of belonging to a specific group (e.g., psychonaut) where respondents share the same experience and have the feeling of being a part of the same community. The feeling of being judged is non-existent, only the social experience is shared together. A second aspect is that respondents testify that their NPS-use reinforces the sociability between members of the group, diminishes the inhibitions, and allow being more voluble. For each groups, NPS use is mostly seen as a part of social context, regardless of the type of substance used, illustrated by the fact that a large majority of users prefers using with a specific group of friends and to share the moment with them.

The fact that it contributes to interaction and discussion, you become very talkative and social. For people with social anxiety, that barrier disappears, they are no longer inhibited and talk to everyone. That’s what attracts me and like I said, I love it when that feeling lasts a long time. (Male, 29)

##### Mind exploration

This category is connected to the concept of looking at things in another way and learning more about oneself. Additional motivations include tempting to approach other levels of the unconsciousness or having a better understanding of their current life situation. Products associated with reaching that state of mind are essentially psychedelics but ketamine can be a mean as well, according to dosage, tolerance, and setting. The lack of coordination provoked by some substances, and the dissociative properties are the determining factors when choosing the location of consumption (e.g., home, friend’s home). Only the occasional and experienced users mentioned this motivation.

I like it because I created my own ‘safe haven’. When I come out of that trip, I have the feeling to have learned so much. The feeling of self-reflection, of increased understanding of the world. Just the feeling that all the pieces of the jigsaw puzzle come together. That I acquired new viewpoints, you know, that very particular doors opened inside my brain that normally remain closed. That is, when being sober or on other substances. (Female, 26)

#### External motivations

##### More convenient use

This category gathers several aspects regarding finding and buying NPS instead of classic illicit substances. Mentioned arguments for buying NPS were as follow: NPS are less expensive than classic illicit drugs, NPS are most often easier to buy especially via channels like the internet or the darkweb; the online market allows to avoid the contact with dealers and the associated shady atmosphere. In addition to increasing the accessibility of NPS, the online market maximizes anonymity as well. Another mentioned reason is the lack of preferred classic illicit substances at that specific time, and as a result, users buy something closely related to their desired first choice substance.

Concerning the quality of NPS, opinions are heterogeneous. Two perspectives are present; if for some users the NPS quality is considered as superior, and therefore diminishes the fear of finding cutting agents, for others the quality is described as inferior to the classic substance. The legal aspect can also be mentioned for a minority of users where the risk or threat of prosecution when using classic illegal substances was mentioned as one of the reasons to prefer buying NPS. Though the underprivileged users are less represented, this motivation was cited by the 3 types of users.

Sometimes I go for what is easiest to get, sometimes I don’t feel like finding another contact to buy speed, a lot of times it’s easier to just have NPS, easy and it’s cheaper as well. Also, sometimes you are just not in the right scene, or you don’t feel like mixing with that scene, finding another shady dealer that you don’t know who is going to give you some speed that weighs half of the original weight after drying and that is of bad quality. For 20€ you can buy a gram of ethylphenidate powder, an analog of rilatine, very high in purity and consistent quality, I receive it in the mail 3 or 4 days after ordering it. And that works very well, at least as good as anything else, it’s more stable, a lot of times it’s just better in fact. (Male, 29)

##### Stronger and specific effects

Looking for specific effects is the major catalysts for choosing and using NPS in this category. Certain NPS are considered to provide stronger effects, or specific effects. Users look for products for which the effects are obtained more quickly and last longer than correlating classic illicit drugs. In addition, users are also interested in the fact that a smaller amount of the product can lead to the same results. Some users also describe the effects of NPS as better effects compared to classic illicit substances; qualifiers such as “much more euphoric” were used. Only the experienced and the underprivileged users named this category.

The effects last a lot longer than those of cannabis. (Male, 31)

#### The negative internal motivation

##### Problematic use

In this class, different notions are interconnected. Above all, the use of NPS is experienced and described by the user himself as a requirement. Using NPS allows users to feel normal, to get up, to go to work, and to deal with the everyday life in a regular way.

Second, NPS can also be used just to get high and forget the life situation or personal issues. Coping, functioning in daily life, and dealing with heath issues, are qualifiers cited as examples by the users. Coping is one of the main functions mentioned among the more underprivileged users. In this group, all respondents confirmed a combining use of classic drugs and NPS, depending on the availability. Classic drugs are, without exception, more important to these users while NPS are merely an expansion of the gamma. NPS substances serve as a coping-mechanism, for yielding confidence and the ability “to not care about what others think.” In addition, NPS serve to coping in an unfriendly world as well, or to be able to assume the familial or job-related responsibilities (e.g., the use of stimulants to study, the use of benzodiazepines to sleep). In a minority of cases, medical considerations play an important role, for example in providing pain relief and in one case mental sanity.

I also am diagnosed with rheumatism. So, I got prescribed Contramal by my GP, which is super heavy. You instantly fall asleep from that stuff. Besides, combined with lots of other substances, it is something far more dangerous than ketamine. So, I’d rather do a key [small amount of ketamine snorted from a key-slot] or two, at home in my couch and be relieved from pain. That is the second function of ketamine for me, next to the psychonautic experiences. (Female, 29)

In addition to the main reasons for using NPS, the following motives were cited: unintentional use, sex, peer pressure, and music/artistic creativity.

## Discussion

As the purpose was to gain a deeper understanding of the culture surrounding the use of NPS, the results demonstrate that a large diversity exists in the motives and reasons for users to consume NPS instead of classic illicit drugs, which is reflected in the large variety in user’s profiles. The reasons for using new substances mainly vary according to personal reasons, regardless of the specific NPS user profile (occasional, more experienced, or even problematic users). Recurring examples include NPS use for pleasure, mind exploration, being connected to others, or out of curiosity. In addition, other structural external reasons are also of influence, such as price, accessibility, purity, or merely for the specific effects procured by certain NPS. Even though the respondents can see the use of NPS as a substitute to classic illicit drugs, choosing NPS for its own effects is also an option (mind exploration, curiosity, specific effects). Our current results on motivations for using NPS are in line with a qualitative study on the users’ perspectives [11]: the data were collected via an online tool among 613 respondents from 42 countries (512 men, 101 women). The following motives were mentioned: more convenient use, curiosity, self-exploration, coping/problematic use, enhanced performance, social bonding/sense of belonging, and pleasure. Another point of interest found in that study and correlating the current results is the fact that motivations to use NPS vary according to the types of NPS and contexts. The results obtained by Barnard et al. [14] also suggest that users tend to have several favorite NPS according to the context of use.

Comparing the results of the study to the motivations for using classic illicit substances, the literature shows that the most cited motivations for using classic illicit drugs are pleasure, energy and enhancement, social bonds, and connections, coping, and self-exploration [11, 31]. These motivational factors are similar as the ones identified here for taking NPS. Studies confirmed also that pleasure or enjoying the effects are the first motivation for using both, classic illicit substances [31] and new psychoactive substances [15, 32]. However, external factors such as price, specific effects, and more convenient use seem to be more specific to the use of NPS [5, 11, 15], as shown in this study as well. As factors of motivation for using classic illicit drugs and NPS tend to overlap, it is important to understand how the NPS consumption is interconnected with classic illicit drug use [14, 33]. Furthermore, based on our results, the use of NPS and classic illicit drugs is usually intertwined; users start with one type of NPS and afterwards try another one, looking for the same or similar kinds of effects procured by classic substances, or looking for something different or new. In that regard, the NPS market has increased the possibilities to find a custom product, with custom mind-altering properties tailored to each specific individual’s preferences. Consequently apprehend the appearance of NPS on the drug market is essential [34].

Among the diversity of motives and contexts of use, the results demonstrate the importance of the social factor as an essential element. Most of the NPS users emphasized the importance of sharing the moment of use as well as the objective of being part of a specific community and connected with others, independently of the type of NPS used. Consequently, using NPS is also seen as a social process shared with friends and allowing a specific connection and bond. Moreover, the online NPS community brought a sense of belonging to users, and a reciprocal sharing. In that regard, this aspect points out the importance of the NPS phenomenon as a social experience [11].

However, based on our results, the problematic use of NPS has to be taken into account as well. It remains essential to understand why the use can become problematic or act as a coping agent that helps overcome the everyday life or relieve pain. By better determining the types of motivations for NPS consumption, NPS-targeted interventions in prevention and harm reduction can be set up or improved [11, 15]. Again, the class of substances used has to be linked to the context and the multiplicity of patterns of use, suggesting that NPS-targeted interventions need to be adapted to socio-cultural characteristics of users (recreational, chemsex), patterns of use, contexts, and their specific risk behaviors (e.g., injecting users) [24, 26].

As the second aim of the study was to provide a comparison of the analytical screenings of the samples provided by the respondents. The results show that a majority of NPS users seem to be quite aware of the identity of the substances they are using. We were not able to detect unexpected hazardous substances in the samples submitted during the study. However, a small proportion of negative results (no active ingredient detected) was found in addition to some hidden additional substances present in several samples. Consequently, the possibility of health risks remains an important possibility. With respect to the definition and the legality of NPS, although a theoretical notion of the concept of newly designed molecules is acquired, identifying a specific substance as an NPS is more complicated for users. The recent results on the NPS *t* study came out with the same conclusion: the NPS term is barely unknown among the users’ population [35]. If users have no idea that they are using NPS, it will be more difficult to target the specific population and therefore to adapt prevention and harm reduction initiatives. The emphasis on the NPS terminology illustrates the complexity in finding an adequate word that is easily understandable by everyone and encompasses the entire diversity of novel substances.

However, the NPS issue should not be seen as a distinct phenomenon, separate from classic illicit substances. On the contrary, similarities with classic illicit drug use have been identified, but differences in pattern of use as well, such as looking for specifics effects and sharing the experiences online with the psychonaut community. Even though NPS has their own specificities, a bigger picture of our modern society that is rapidly changing in terms of technological possibilities is necessary to understand NPS as the next chapter in the history of drug use (including classic and novel substances).

## Conclusion

To conclude, when determining which types of NPS-targeted interventions are adapted to the different user profiles and hence need implementation, a good understanding of the type of motivations and the patterns of NPS use, together with the culture surrounding the use of NPS is important. In that regard, the interconnections between the use of NPS and classic illicit drugs deserves further investigation in the health and social fields.

## Limitations

Despite the methodology used to reach the target populations and the use of incentives, this study has limitations. Indeed, even though a diversity of the study sample is present and gives us a first overview on the profiles of NPS users in Belgium, we did not reach every hidden groups.

## Data Availability

The datasets generated and/or analyzed during the current study are not publicly available due to individual privacy but are available from the corresponding author on reasonable request.

## Abbreviations

BEWSD: Belgian early warning system drugs
EMCDDA: European Monitoring Centre for Drugs and Drug Addiction
MSM: Men having sex with men
NPS: New psychoactive substances
NPSMM: New psychoactive substance use motives measures
